# ‘Reducing anxiety and maintaining care’ during the COVID-19 pandemic

**DOI:** 10.1093/rap/rkaa062

**Published:** 2020-10-28

**Authors:** Polly Livermore

**Affiliations:** Rheumatology Matron and Clinical Academic Lead, NIHR GOS BRC, Rheumatology Department, Great Ormond Street Children’s Hospital, London, UK

**Key message**

• The paediatric rheumatology nurse has been instrumental in maintaining care and reducing anxiety during the pandemic.

Dear  Editor, In March of this year, overnight the care of rheumatology patients changed significantly. For both adult and paediatric patients, severe restrictions were put into place, and families found that they could not access their usual services and treatments. At the height of the pandemic, immunocompromised children and young people receiving named medications at certain doses, were encouraged to ‘shield’. This advice caused a tsunami of parents calling paediatric rheumatology services to ask for advice on their child. Parents asked questions about shielding, stopping therapies, changing therapies, appointments being cancelled, clinics being changed to virtual, school attendance and many, many more. More often than not, the first port of call for these concerns was the paediatric rheumatology nurse specialist. However, with the variation around the UK in how national guidelines were developed and implemented and with the constantly changing advice that was being cascaded, staying on top of all this information and having enough nurses to disseminate this guidance safely has been a challenge.

The British Society of Rheumatology (BSR) State of Play report [1] emphasizes that rheumatology nurse specialists are at the forefront of patient care. Paediatric rheumatology nurse specialists, in particular, have a dual role, not only managing the care of children, but also the inherent parental anxiety. During the worldwide pandemic, we were interested to know how this role has adapted to meet the current needs and concerns of paediatric patients and their families. 

As a key nurse representative for the British Society for Rheumatology for the United Kingdom (UK), I distributed an electronic survey to an established active email group of paediatric rheumatology nurses across the UK. This email group is known to include the majority of paediatric rheumatology nurses around the UK who have paediatric rheumatology nursing as their main role. Nevertheless, it must be acknowledged that there are some nurses who care for paediatric rheumatology patients combined with other specialty paediatric caseloads or those who are predominantly adult nurses, which are not part of this group, thought to be ∼10–15 nurses. The survey was structured in three sections: information about the individual, information about their role during the pandemic and their concerns for the future. Ethical considerations were paramount; the respondents were assured of confidentiality of their responses, no personal data were requested, and return of the survey was taken as assent to take part.

The survey was sent to 60 paediatric rheumatology nurses in July 2020, with 50 (83%) responding from the majority of secondary and tertiary centres. The biggest proportion were Band 6 Clinical Nurse Specialists (*n* = 18, 36%), followed by Band 7 Clinical Nurse Specialists (*n* = 16, 32%), and 46 of these (86%) were members of BSR. The majority (*n* = 18, 36%) have worked in paediatric rheumatology for between 2 and 5 years, with five (10%) new to the service (<3 months) and at the other extreme, four (8%) have been working for >20 years in the speciality. Of these 50, 15 (30%) are independent nurse prescribers, and only 8 of these (16%) administer intra-articular CS joint injections. 

When considering their role during the pandemic, 17 (34%) respondents were expecting to be redeployed, but only 7 (14%) were redeployed. Of these seven, only one was moved to an area in which they were previously employed (the high-dependency unit), whereas the other six (12%) were moved to areas they had not worked in previously.

All 50 respondents (100%) said that their roles had changed since the pandemic began, with 36 (72%) nurses saying they had been allowed to work from home and 26 (52%) who were involved in delivering virtual clinics.

**Fig. 1:**
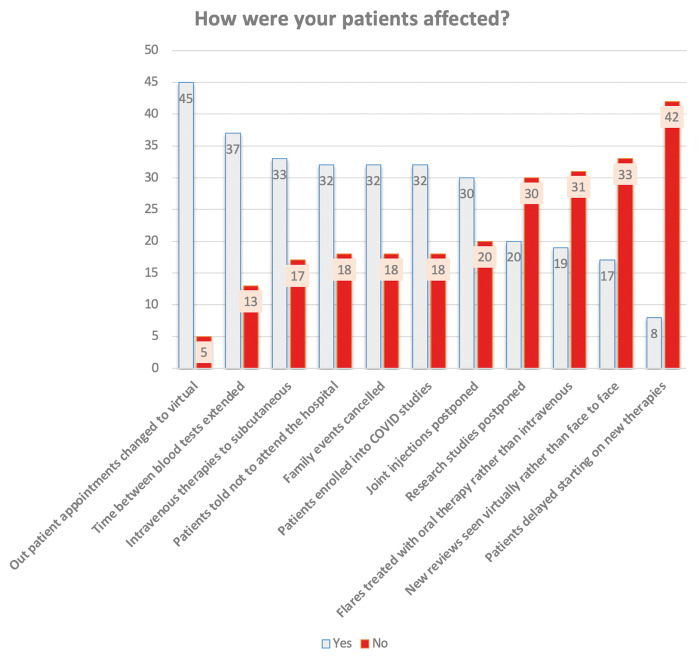
Graph showing how paediatric rheumatology patients were affected during the recent coronavirus disease-2019 pandemic COVID-19: coronavirus disease-2019.

All 50 respondents agreed that patient care had also changed, as illustrated further by Fig. 1. The majority (*n* = 48, 96%) were regularly giving shielding advice, 33 (66%) changed patients from IV hospital-based therapy to home administration and 30 (60%) said that IA joint injections had been postponed. Reducing anxiety and maintaining care were described as key daily roles.

Nurses provided extra information in their qualitative comments, such as:

We have done fewer home visits.

Patients stopped medication as they were frightened due to immunosuppression and not telling us.

We have not stopped treatments on stable patients if they were due to stop. This was to avoid possible flare requiring possible hospital admission.

No general anaesthetic (GA) joint injections carried out as they would require 14 day family isolation pre GA.

Lots of virtual professionals meetings too. Maintaining what is going on and keeping on top of things is very challenging. Patient anxiety has significantly added to calls and emails to service.

The UK nursing workforce has made a substantial contribution during the coronavirus disease-2019 (COVID-19) public health emergency [2]. However, although the current focus is on getting through the pandemic, the hardest challenge might be to return to a sense of normality and fix some of the inevitable consequences, such as:

Patients with delayed joint injections have now developed fix flexion deformities.

Re-establishing care is even more challenging than cancelling.

The recently published ‘Paediatric and Adolescent Rheumatology – The State of Play’ [3] clearly highlights the high vacancy rates of Paediatric Rheumatology Clinical Nurse Specialists. Without retaining this current skilled and expert workforce, who have clearly demonstrated their worth during this pandemic, we could be at risk of loss of specialized and experienced care, putting our patients at risk. This survey provides a snapshot of the changing and challenging role of the nurse specialist during current times and highlights the importance and adaptability of their role within the rheumatology team.
